# Effects of clonal integration and nutrient availability on the growth of *Glechoma longituba* under heterogenous light conditions

**DOI:** 10.3389/fpls.2023.1182068

**Published:** 2023-08-15

**Authors:** Rui Zhang, Zhi-Huan Chen, Yu-Meng Li, Ning Wang, Wen-Tao Cui, Bing-Nan Zhao, Chao Si

**Affiliations:** ^1^ School of Life Science and Engineering, Handan University, Handan, China; ^2^ School of Special Education, Handan University, Handan, China

**Keywords:** clonal integration, *Glechoma longituba*, heterogeneity, light condition, nutrient availability

## Abstract

**Introduction:**

Clonal integration of connected ramets within clones is an important ecological advantage. In this study, we tested the hypothesis that the effects of clonal integration on performance of donor and recipient ramets when one resource is heterogeneous can be influenced by the availability of another resource of donor ramets.

**Methods:**

We conducted a greenhouse experiment on the widespread, perennial herb *Glechoma longituba*. Clonal fragments consisting of pairs of connected ramets were grown for seven weeks. The younger, apical ramets were exposed under 30% or 100% light condition and the older, basal ramets were treated with three levels of nutrients. The connections between ramets were either severed or left intact. 30% light condition negatively affected the growth of apical ramets, basal ramets and the whole fragments.

**Results:**

Clonal integration significantly increased the growth of apical ramets, but decreased the growth of the basal ramets. Medium and high level nutrient availability of basal ramets significantly increased the growth of apical ramets, basal ramets and the whole fragments. At the high nutrient level, the reduction in growth of basal ramets from clonal integration was decreased, but the growth responses of apical ramets and the whole fragments to clonal integration were not influenced by nutrient availability.

**Conclusion:**

The results suggested that clonal integration was benefit to the growth of apical ramets of Glechoma longituba but at the cost of reducing the growth of basal ramets. Although the high nutrient level could reduce the cost that clonal integration brought to the unshaded basal ramets, but could not increase the benefit that clonal integration brought to the shaded apical ramets and whole fragment.

## 1 Introduction

Clonal plants are common in many natural habitats and are often the dominant species (Prach and Pyšek, 1994; Evans and Cain, 1995; Liu et al., 2007). The clonal growth of plants mainly involves the production of vegetative offspring that remain connected to the parent by connective architectures (e.g., stolons, rhizomes, or horizontally growing roots), which form a large network (Prach and Pyšek, 1994; Alpert, 1996; Gao et al., 2012). Among interconnected ramets, water, mineral nutrients, photosynthates, and chemical signals can be translocated via the connective architecture, that is, clonal integration (Caraco and Kelly, 1991; Alpert, 1996). Clonal integration is a uniquely advantageous trait for clonal plants (Hutchings and Kroon, 1994; Hutchings and Wijesinghe, 1997; Kroon and Groenendael, 1997). During the establishment of offspring (or distal) ramets, parent (or proximal) ramets can provide resources through clonal integration to help them successfully colonize and quickly spread (Zhang et al., 2003; Elgersma et al., 2015).

The distribution of resources such as nutrients, light, and water in natural environments is heterogeneous (Ricklefs, 1977; Caldwell and Pearcy, 1994; Stein et al., 2014). That is, levels of resources are consistent within the same patch but inconsistent among different patches (Stuefer and Hutchings, 1994; Qian et al., 2014). Resource heterogeneity can influence plant growth, intraspecific and interspecific competition, and community structures (Roiloa et al., 2014; Wan et al., 2019; Liang et al., 2020; Si et al., 2021).

In natural habitats, clonal plants usually occupy a large area and inevitably span patches with different resource levels (Roiloa and Retuerto, 2007; Xing et al., 2019). Clonal integration plays an important role in the distribution of connected ramets in heterogeneous environments (Glover et al., 2015; Zhang et al., 2016; Si et al., 2020a). Ramets in patches with higher recourses (i.e., donor ramets) can transfer resources to connected ramets in patches with lower recourses (i.e., recipient ramets) (Zhang et al., 2016).

Furthermore, when resources are translocated among connected ramets, there is a cost–benefit relationship between donor and recipient ramets (van Kleunen and Stuefer, 1999; Zhang et al., 2015; Si et al., 2020a). If the benefit of recipient ramets is greater than the cost of donor ramets, the whole clone will benefit. Otherwise, the growth of the entire clone is consumed (Chen et al., 2010; Gao et al., 2013; Zhang et al., 2015). If the benefit is consistent with the cost, overall growth will be consistent (Adomako et al., 2020; Si et al., 2020a). However, whether the cost–benefit relationship between connected ramets is affected by another resource when they grow in a heterogeneous environment is limited.

To occupy more space, offspring ramets of dwarf clonal plants usually spread to the shaded environment caused by coexisting tall trees or shrubs that block the light (Slade and Hutchings, 1987; Li et al., 2018a; Li et al., 2018b). Previous studies have shown that the growth of shaded ramets can be improved using unshaded ramets through clonal integration (Li et al., 2018a; Si et al., 2020b). Nutrient availability is a key factor affecting plant performance (Lebauer and Treseder, 2008; Song et al., 2015; Gao et al., 2021). An increase in nutrient availability can promote plant growth and change the population dynamics of the plant (Si et al., 2019; Zhang et al., 2020; Zhang et al., 2022). Previous studies have indicated that ramets with low nutrient availability and whole plants could benefit from clonal integration in heterogeneous nutrient environments (Zhang and He, 2009; You et al., 2014; Wang et al., 2017). We hypothesized that nutrient availability in unshaded ramets can influence the effects of clonal integration on plant performance.

To test this hypothesis, we conducted a control experiment in a greenhouse. In the experiment, fragments of the widespread, herbaceous, clonal species *Glechoma longituba* were planted in pairs in pots. The basal ramets grow unshaded and receive three levels of nutrients, whereas the apical ramets are shaded. Stolons between the basal and apical ramets were either severed or left intact. Specifically, we tested the following hypotheses: (1) shading can decrease the growth of apical ramets, (2) clonal integration can increase the growth of apical ramets, and (3) the high nutrient availability of the unshaded basal ramets can enhance the benefits of clonal integration brought to the shaded apical ramets and whole fragments.

## 2 Materials and methods

### 2.1 Plant species

*G. longituba* L. is a perennial clonal herb belonging to the Lamiaceae family (Chu et al., 2006; Zhang et al., 2007; Zhang and He, 2008). This species is common in forests, roadsides, and creeks, and is distributed throughout China, except for the Northwest and Inner Mongolia Autonomous Region (Zhang et al., 2007). The monopodial stolon extends and forms a network on the ground (Chu et al., 2006). Along the stolon, each node can produce two opposite zygomorphic single leaves and adventitious roots, thereby functioning as a potentially independent ramet. Every leaf axil bears one bud, which may grow into a secondary stolon (Chu et al., 2006; Zhang et al., 2007). The experimental plant materials were purchased from a commercial supplier in Shanghai, China. The plants were cultivated for several weeks in a greenhouse (36°34′N, 114°29′E) at Handan University in Handan, Hebei Province, China, before the commencement of the experiment.

### 2.2 Experimental design

**Figure 1:**
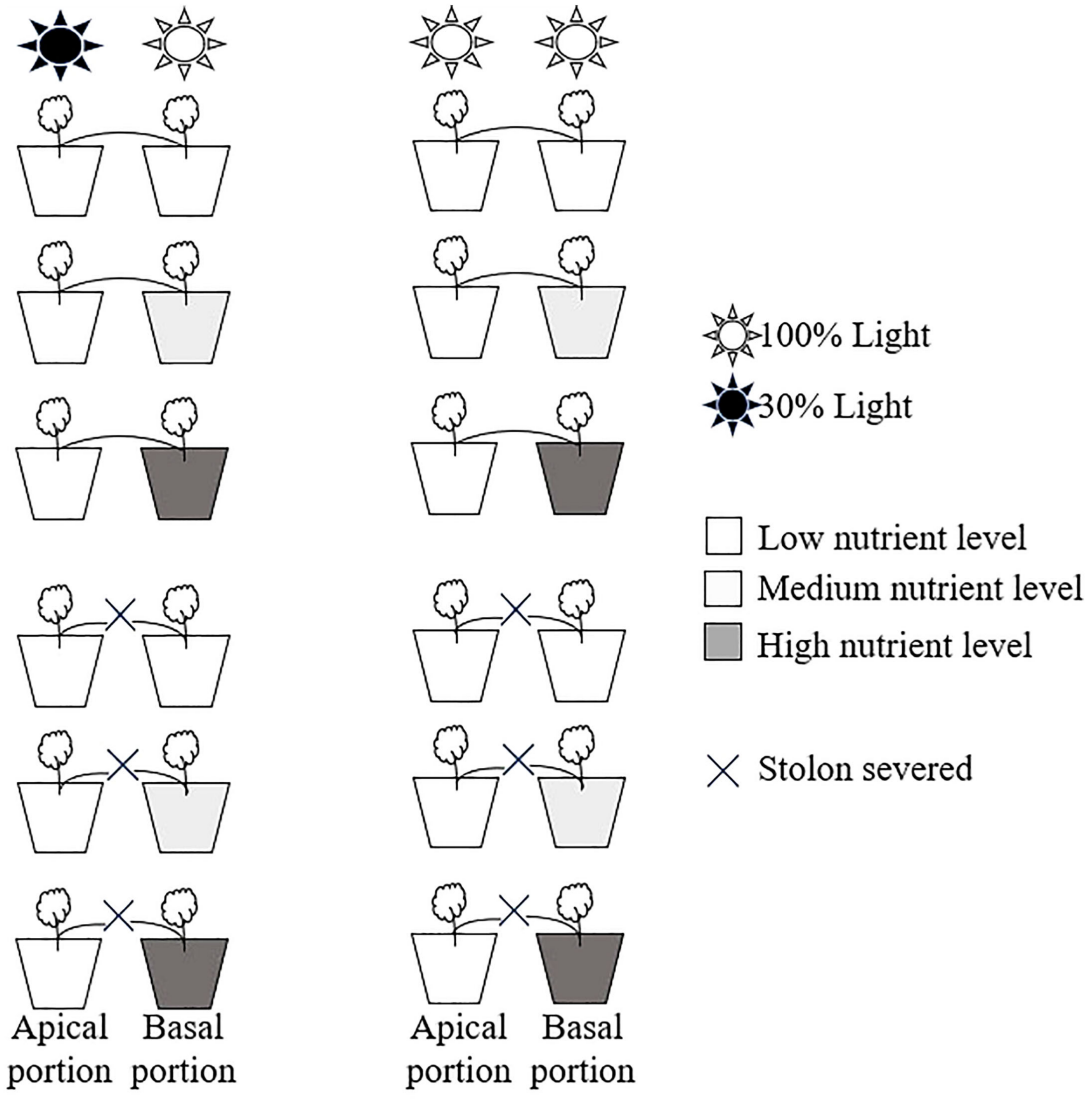
Schematic representation of the experimental design. Apical portions of *Glechoma longituba* grew under 30% or 100% light conditions. Basal portions were grown at three nutrient levels under 100% light conditions. The stolon between the apical and basal portions was either severed or left intact.

The experiment had two levels of clonal integration treatments (stolon severed or stolon left intact) fully crossed with two levels of light intensity (apical ramets grown under shaded conditions or under unshaded conditions) and three levels of nutrient availability (basal ramets grown under low, medium, or high nutrients conditions) (**Figure 1**).

On 22 April 2021, 84 ramets bearing a pair of fully expanded leaves were severed from stock plants. These ramets was planted on the potted trays which were filled with equal volumes of potting soil (0.17 g total N kg^−1^, 2.1 mg total P kg^−1^, and 1.5 g total K kg^−1^; Dewoduo Fertilizer Co., China) and river sand. After 36 days, 60 plants in which the ramet had produced a new axillary stolon at least 15 cm long, with four nodes and an apex, were selected and used in this experiment.

The three proximal nodes, referred to as the basal portion, were allowed to root in the pots (15 cm in diameter and 13 cm in depth) which were filled with a mixture of the local collected topsoil (0.83 g total N kg^−1^ and 20.37 g total C kg^−1^), and peat (particle size <6 mm; Klasmann, Germany) at a volume ratio of 1:1. One distal node and the apex, referred to as the apical portion, were rooted in a separate, same pot that was filled with the same mixture as the basal one. The apical portions were placed under ambient light conditions or shaded to 30% ambient light with a shade cloth. For the three levels of nutrient treatments in the basal portions, the pots received 300 mL of 0, 0.2, or 1 g water-soluble fertilizer (Peters Professional 20-20-20+TE General Purpose Fertilizer, Everris, NA, Inc.: 20% total N, 20% available PO_4_, and 20% soluble potash) L^−1^ tap water every 10 days. When the basal portions received nutrient solution, tap water was provided to the apical portions of the same volume. In the severed treatment, the stolon connecting the basal and apical portions was cut on 17 June 2021. In the clonal integration treatment, these portions were left intact. Each treatment was replicated five times, resulting in a total of 60 pairs of pots. All pot pairs were placed randomly on a bench in the same greenhouse for cultivation.

Plants were allowed to grow for 7 weeks. During the experiment, the daily mean temperature and relative humility were 28.9 °C and 50.2%, respectively. New stolons and ramets produced by each portion of each fragment were allowed to root in the same pot as the rest of the fragments.

### 2.3 Measurements and data analysis

We harvested the experiment when the plants covered the entire soil surface in many containers. At harvest, we counted the number of nodes in *G. longituba* and measured total stolon length in each pot. Then, the plants were separated into leaves, stolons, and roots, dried at 70 °C to a constant weight, and weighed. We also calculated the specific stolon length (total stolon mass/total stolon length) and root–shoot ratio [root mass/(leaf mass + stolon mass)].

We used three-way ANOVA to test the effects of clonal integration, light conditions, and nutrient availability on total mass, leaf mass, stolon mass, root mass, node number, total stolon length, specific stolon length, and root–shoot ratio in the apical portion, basal portion, and whole clonal fragment. Data were checked for normality and homoscedasticity and transformed before analysis, as needed, to improve homoscedasticity. Total mass, leaf mass, stolon mass, root mass, node number, specific stolon length of the apical portion, leaf mass, node number of the basal portion, total mass, leaf mass, stolon mass, root mass, total stolon length, and root–shoot ratio of the whole fragment were transformed into square roots. The total stolon length and root–shoot ratio of the apical portion, total mass, stolon mass, root mass, total stolon length, root–shoot ratio of the basal portion and node number of the whole fragment were transformed to the natural log. Figures show untransformed data. All analyses were conducted using SPSS 22.0 (IBM, Inc., Armonk, NY, USA).

## 3 Results

### 3.1 Effects of clonal integration, light condition, and nutrient availability on apical portions

**Figure 2:**
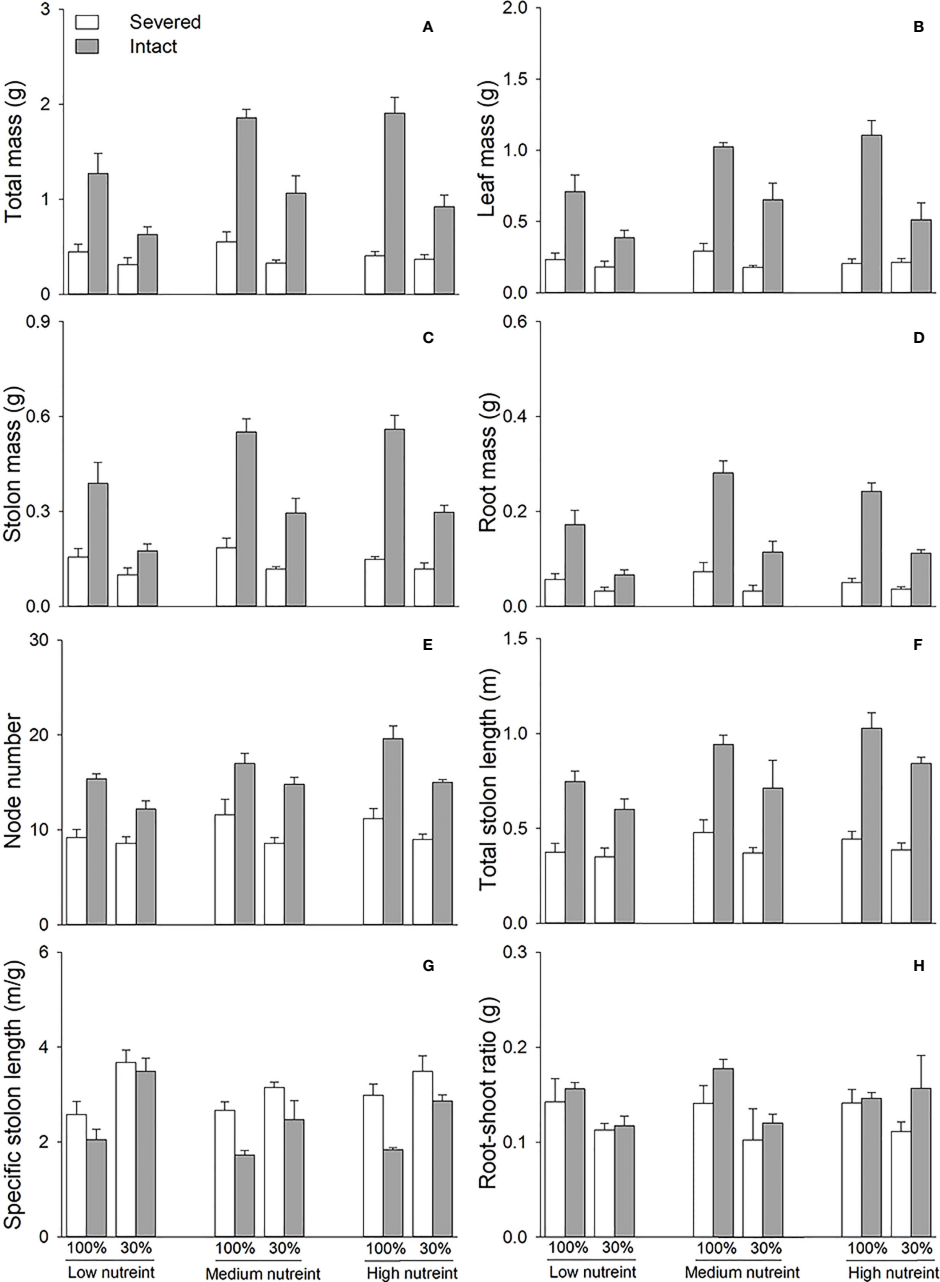
Total mass **(A)**, leaf mass **(B)**, stolon mass **(C)**, root mass **(D)**, node number **(E)**, total stolon length **(F)**, specific stolon length **(G)**, and root–shoot ratio **(H)** of the apical portions of *Glechoma longituba* when the apical portions were under 30% or 100% light conditions, the basal portions at low, medium, or high nutrient levels, and the connection between the two portions were either severed or left intact. Bars and vertical lines represent the mean and SE (n = 5).

Clonal integration significantly increased the biomass (total mass, leaf mass, stolon mass, and root mass), node number, total stolon length, and root–shoot ratio of the apical portions of *G. longituba*, but significantly decreased the specific stolon length (**Table 1**; **Figure 2**). Biomass, total stolon length, and root–shoot ratio were significantly decreased under 30% light conditions, while the response of specific stolon length was the opposite (**Figures 2A–D**). Nutrient availability significantly affected the biomass, node number, total stolon length, and stolon length (**Table 1**). Medium and high levels of nutrients increased biomass, node number, and total stolon length of apical portions, while the specific stolon length decreased (**Figures 2A**). The interaction between clonal integration and light conditions significantly affected the biomass of the apical portions. Compared to the 30% light condition, the increase in biomass was greater under 100% light conditions when the stolon was left intact (**Table 1**; **Figures 2A**). Clonal integration and nutrient availability had a significant interaction effect only on the stolon mass of the apical portion. Specifically, the increase in stolon mass by clonal integration was greater at medium- and high-nutrient levels than at low-nutrient levels (**Table 1**; **Figure 2**).

**Table 1:** Effects of clonal integration, light condition, and nutrient availability on total mass, leaf mass, stolon mass, root mass, node number, total stolon length, specific stolon length, and root–shoot mass ratio of the apical portions of *Glechoma longituba*.

Variable	Integration (I)		Light (L)		Nutrient (N)		I × L		I × N		L × N		I × L × N	
	F_1,48_	*P*	F_1,48_	*P*	F_2,48_	*P*	F_1,48_	*P*	F_2,48_	*P*	F_2,48_	*P*	F_2,48_	*P*
Total mass^a^	**170.1**	**<0.001**	**40.2**	**<0.001**	**5.7**	**0.006**	**13.4**	**0.001**	3.1	0.058	0.1	0.914	0.7	0.490
Leaf mass^a^	**128.2**	**<0.001**	**23.7**	**<0.001**	**3.6**	**0.035**	**10.7**	**0.002**	1.9	0.160	0.2	0.859	1.5	0.227
Stolon mass^a^	**161.9**	**<0.001**	**53.1**	**<0.001**	**7.3**	**0.002**	**13.1**	**0.001**	**3.6**	**0.036**	0.1	0.912	0.1	0.918
Root mass^a^	**139.3**	**<0.001**	**53.9**	**<0.001**	**4.4**	**0.017**	**12.4**	**0.001**	3.1	0.056	0.9	0.405	<0.1	0.955
Node number^a^	**123.3**	**<0.001**	**21.7**	**<0.001**	**6.1**	**0.004**	0.7	0.412	0.9	0.412	0.4	0.652	1.0	0.365
Total stolon length^b^	**101.4**	**<0.001**	**8.6**	**0.005**	**4.3**	**0.019**	0.8	0.371	0.7	0.492	0.5	0.605	0.1	0.936
Specific stolon length^a^	**27.4**	**<0.001**	**41.4**	**<0.001**	**3.6**	**0.034**	2.7	0.104	1.6	0.209	1.8	0.169	0.2	0.854
Root–shoot ratio^b^	**5.9**	**0.019**	**11.4**	**0.001**	0.3	0.768	0.1	0.718	0.6	0.558	1.3	0.274	0.5	0.637

^a^Square root transformation. ^b^Natural log transformation. Degree of freedom (subscript for ‘‘F’’), F and *P*-values are given. Values are in bold when *P <0.05*.

### 3.2 Effects of clonal integration, light condition, and nutrient availability on basal portions

**Figure 3:**
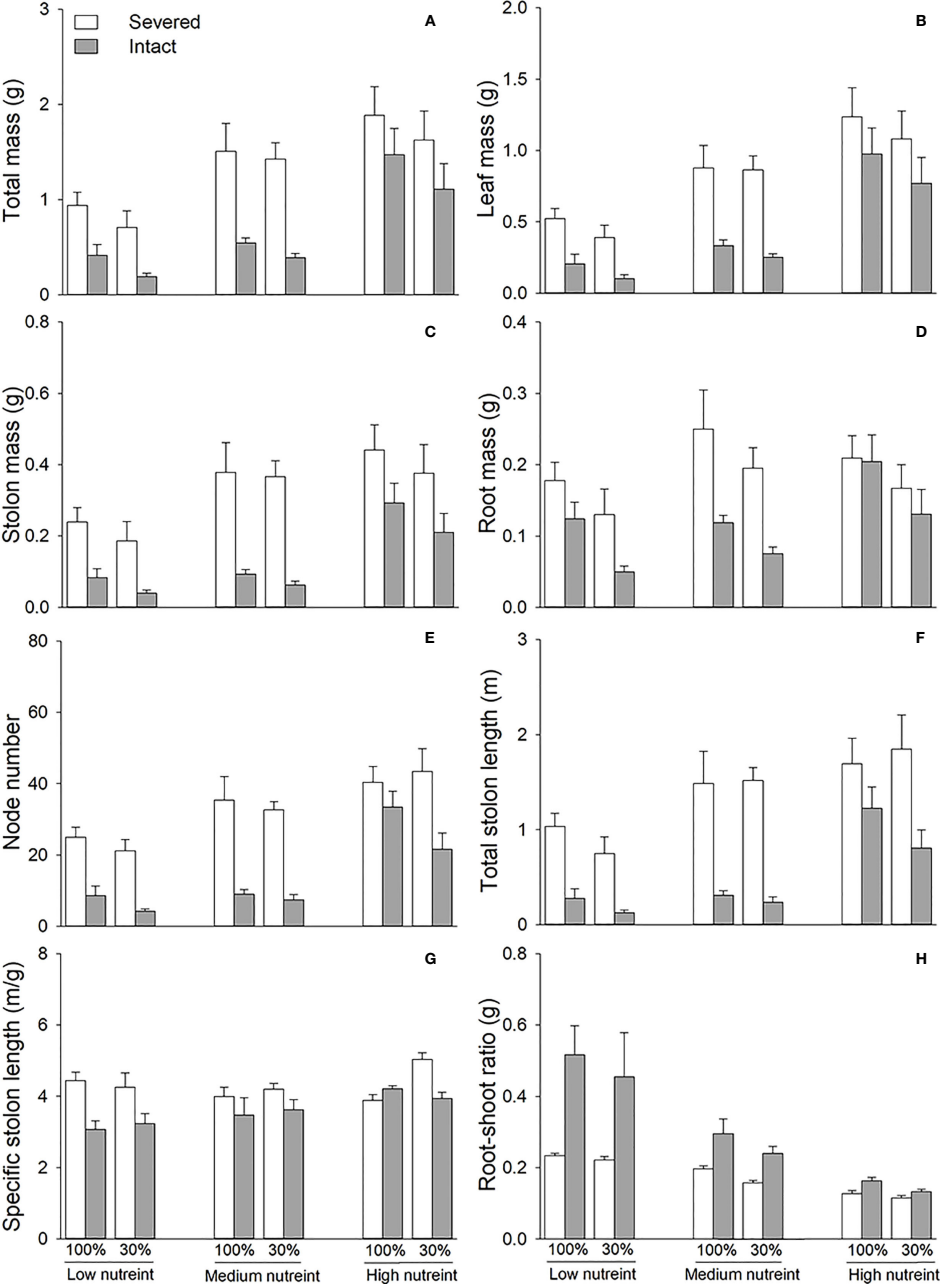
Total mass **(A)**, leaf mass **(B)**, stolon mass **(C)**, root mass **(D)**, node number **(E)**, total stolon length **(F)**, specific stolon length **(G)**, and root–shoot ratio **(H)** of the basal portions of *Glechoma longituba* when the apical portions were under 30% or 100% light conditions, the basal portions at low, medium, or high nutrient levels, and the connection between the two portions were either severed or left intact. Bars and vertical lines are means and SE (n = 5).

Clonal integration significantly decreased the biomass, node number, total stolon length, and specific stolon length of the basal portions of *G. longituba* but increased the root–shoot ratio (**Table 2**; **Figure 3**). The 30% light condition decreased biomass, node number, and total stolon length but the increased root–shoot ratio (**Figures 3A–F**). Increased nutrient availability increased biomass, node number, total stolon length, and specific stolon length but decreased the root–shoot ratio (**Figure 3**). The interaction between clonal integration and nutrients significantly affected biomass, node number, total stolon length, and root–shoot ratio (**Table 2**). The decrease in biomass, node number, and total stolon length under clonal integration was lower at high nutrient levels, and the increase in the root–shoot ratio was reduced (**Figures 3A–F**).

**Table 2:** Effects of clonal integration, light condition, and nutrient availability on total mass, leaf mass, stolon mass, root mass, node number, total stolon length, specific stolon length, and root–shoot mass ratio of the basal portions of *Glechoma longituba*.

Variable	Integration (I)		Light (L)		Nutrient (N)		I × L		I × N		L × N		I × L × N	
	F_1,48_	*P*	F_1,48_	*P*	F_2,48_	*P*	F_1,48_	*P*	F_2,48_	*P*	F_2,48_	*P*	F_2,48_	*P*
Total mass^b^	64.4	**<0.001**	**8.8**	**0.005**	**39.0**	**<0.001**	1.8	0.187	**6.1**	**0.004**	1.0	0.377	0.1	0.885
Leaf mass^a^	46.6	**<0.001**	**4.1**	**0.048**	**45.0**	**<0.001**	0.3	0.576	3.1	0.056	0.3	0.714	<0.1	0.969
Stolon mass^b^	86.6	**<0.001**	**7.1**	**0.011**	**26.0**	**<0.001**	1.8	0.191	**7.0**	**0.002**	0.6	0.567	0.1	0.917
Root mass^b^	29.0	**<0.001**	**20.6**	**<0.001**	**7.3**	**0.002**	2.9	0.094	**3.8**	**0.028**	0.8	0.429	0.2	0.819
Node number^a^	108.3	**<0.001**	**4.3**	**0.045**	**37.4**	**<0.001**	2.1	0.150	**5.5**	**0.007**	0.3	0.774	0.8	0.455
Total stolon length^b^	115.3	**<0.001**	**4.9**	**0.032**	**33.0**	**<0.001**	2.5	0.118	**9.0**	**<0.001**	0.9	0.418	0.1	0.928
Specific stolon length	21.3	**<0.001**	1.8	0.192	**4.3**	**0.018**	1.5	0.227	2.6	0.085	0.7	0.483	3.0	0.059
Root–shoot ratio^b^	43.7	**<0.001**	**6.7**	**0.013**	**63.6**	**<0.001**	0.3	0.560	**4.6**	**0.015**	0.1	0.916	0.2	0.801

^a^Square root transformation. ^b^Natural log transformation. Degree of freedom (subscript for ‘‘F’’), F and *P*-values are given. Values are in bold when *P <0.05*.

### 3.3 Effects of clonal integration, light condition, and nutrient availability on the whole clonal fragments

**Figure 4:**
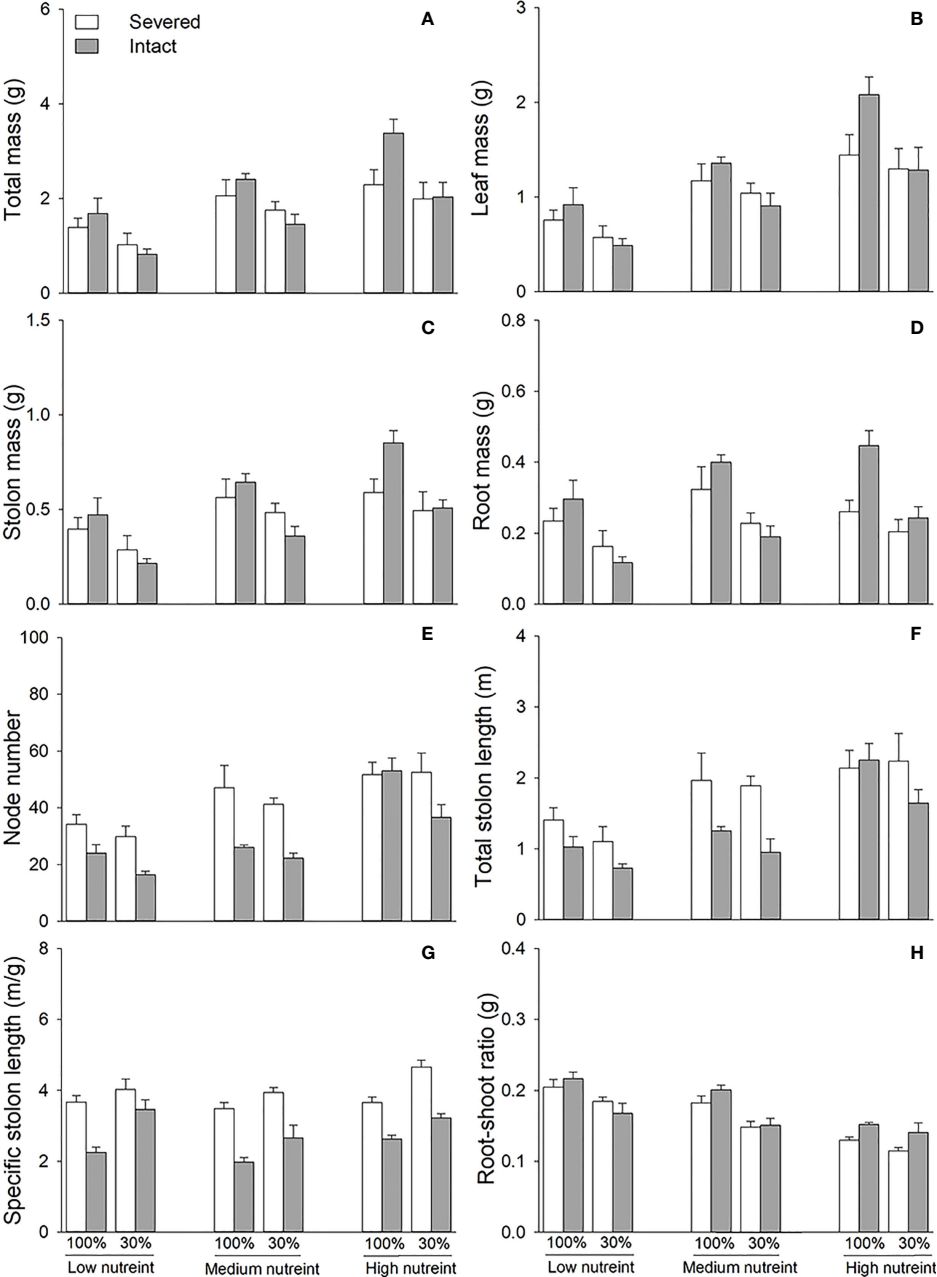
Total mass **(A)**, leaf mass **(B)**, stolon mass **(C)**, root mass **(D)**, node number **(E)**, total stolon length **(F)**, specific stolon length **(G)**, and root–shoot ratio **(H)** of whole fragments of *Glechoma longituba* when the apical portions were under 30% or 100% light conditions, the basal portions at low, medium, or high nutrient levels, and the connection between the two portions either severed or left intact. Bars and vertical lines are means and SE (n = 5).

Clonal integration significantly decreased the node number, total stolon length, and specific stolon length but increased the root–shoot ratio of the whole fragment of *G. longituba* (**Table 3**; **Figures 4E**). The 30% light condition significantly decreased biomass, node number, total length, specific stolon length, and root–shoot ratio (**Figure 4**). Biomass, node number, total stolon length, and specific stolon length increased, but the root–shoot ratio decreased with an increase in nutrient availability (**Figure 4**). The interaction between clonal integration and light conditions significantly affected the biomass (**Table 1**). Under 100% light conditions, clonal integration increased the biomass of the whole fragments. Under 30% light conditions, biomass decreased (**Figures 4A**). The interaction between clonal integration and nutrient availability significantly affected the node number of whole fragments (**Table 3**). The reduction in the number of nodes by clonal integration decreased when the nutrient levels were high (**Figure 4**).

**Table 3:** Effects of clonal integration, light condition, and nutrient availability on total mass, leaf mass, stolon mass, root mass, node number, total stolon length, specific stolon length, and root–shoot mass ratio of all *Glechoma longituba* fragments.

Variable	Integration (I)		Light (L)		Nutrient (N)		I × L		I × N		L × N		I × L × N	
	F_1,48_	*P*	F_1,48_	*P*	F_2,48_	*P*	F_1,48_	*P*	F_2,48_	*P*	F_2,48_	*P*	F_2,48_	*P*
Total mass^a^	1.3	0.255	**21.6**	**<0.001**	**22.6**	**<0.001**	**5.4**	**0.025**	1.0	0.358	0.1	0.932	0.1	0.927
Leaf mass^a^	1.2	0.284	**15.4**	**<0.001**	**28.4**	**<0.001**	**4.5**	**0.040**	0.7	0.518	0.1	0.863	0.2	0.834
Stolon mass^a^	0.6	0.427	**25.4**	**<0.001**	**17.4**	**< 0.001**	**5.9**	**0.018**	1.5	0.241	0.1	0.922	0.1	0.947
Root mass^a^	3.5	0.068	**39.9**	**<0.001**	**7.6**	**0.001**	**7.1**	**0.010**	2.2	0.127	0.1	0.868	<0.1	0.999
Node number^b^	**47.9**	**<0.001**	**10.6**	**0.002**	**39.7**	**<0.001**	3.6	0.064	**4.6**	**0.015**	0.4	0.689	0.5	0.600
Total stolon length^a^	**16.2**	**<0.001**	**5.2**	**0.028**	**22.8**	**<0.001**	1.8	0.182	2.4	0.102	0.1	0.866	0.5	0.638
Specific stolon length	**104.6**	**<0.001**	**37.3**	**<0.001**	**6.9**	**0.002**	0.9	0.337	1.0	0.380	0.4	0.680	2.4	0.105
Root–shoot ratio^a^	**4.6**	**0.037**	**32.7**	**<0.001**	**45.0**	**<0.001**	1.5	0.226	2.9	0.067	2.2	0.117	0.8	0.438

^a^Square root transformation. ^b^Natural log transformation. Degree of freedom (subscript for ‘‘F’’), F and *P*-values are given. Values are in bold when *P <0.05*.

## 4 Discussion

The low light intensity caused by shading significantly reduced the growth of apical ramets in *G. longituba*, probably because of the reduced photosynthetic rate (Madsen and Adams, 1989; Everitt and Burkholder, 1991; Wang et al., 2012). Moreover, plants can adjust themselves to adapt to low-light environments (Cronin and Lodge, 2003; Going et al., 2008; Si et al., 2020b). In this experiment, apical ramets produced shorter and thinner stolons under low-light conditions, which is inconsistent with the response observed in many other terrestrial clonal plants (Barko and Filbin, 1983; Maegawa et al., 1987; Tavechio and Thomaz, 2003). We attributed these differences to the variation in the trade-off between the cost of respiration and the benefits of photosynthesis across clonal plant species. The root–shoot ratio of apical ramets decreased under 30% light conditions. This may be a positive adaptive strategy for plants, as explained in the typical resource-ratio hypothesis, i.e., plants tend to allocate more biomass to aboveground organs to compete for light when light is limited (Zhou et al., 2012; Xie et al., 2016; Yu et al., 2018).

As expected, clonal integration significantly increased the growth of apical portions under shaded conditions, although this increase was lower than that when the apical portions were not shaded. Meanwhile, the growth of the basal portions decreased when the stolon was left intact, and the performance of whole fragments was not significantly influenced by clonal integration. The cost–benefit relationship between donor and recipient ramets was inconsistent in previous studies (Chen et al., 2010; Roiloa and Retuerto, 2012; Si et al., 2020b). For instance, clonal integration improved the performance of *Fragaria vesca* offspring grown in copper-polluted soil but reduced the photosynthetic efficiencies and growth of their parent (as the donor) (Roiloa and Retuerto, 2012). In another study, although clonal integration brought benefits to distal ramets (as the recipient) of *Carex praeclara* buried in sand, the cost was not detected in proximal ramets (as the donor) (Chen et al., 2010). The results of this experiment demonstrated that the benefits of clonal integration to apical ramets of *G. longituba* may be at the cost of reducing the growth of basal ramets, regardless of the light condition of apical ramets and the nutrient availability level of basal ramets.

The increase in nutrients improved the growth of the apical ramets, basal ramets, and whole fragments, but did not enhance the benefits of clonal integration brought to apical ramets and whole fragments as predicted. However, there is a noteworthy finding in this experiment that the reduction in basal ramets by clonal integration was significantly reduced at high nutrient levels. These results suggest that a higher level of nutrient availability can reduce the consumption of basal ramets caused by clonal integration. In addition, biomass allocation of basal ramets responded significantly to nutrient levels when clonal integration was maintained. With the increase in nutrients, the biomass invested in the roots of basal ramets was less when the stolon was intact than when it was severed. This may be because basal ramets need to invest more biomass in the roots to ensure their nutrient uptake and supply to apical ramets when nutrient availability is low. When nutrient levels are high, they invest more biomass in the aboveground organs to improve photosynthesis (Wang et al., 2008). This allocation strategy may maximize the performance of the whole clone by preferentially acquiring and using resources that are more valuable (Roiloa and Retuerto, 2007). A limitation of this study is that we defined the nutrient level of the soil itself as a low nutrient level, but for apical ramets, this nutrient level may be a level that can support plant growth. We do not deny that the results may be different when nutrient levels are lower or higher than those used in this experiment. In addition, this experiment lasted for only 7 weeks; if the plant is allowed to grow longer, the response of the plant may change accordingly.

## 5 Conclusion

Limited light conditions of the apical ramets negatively affected the growth of the apical portions of *G. longituba*. Clonal integration was beneficial to the growth of apical portions but was a consumption of basal portions. The benefit of clonal integration to the shaded apical portions did not improve when the nutrient availability of the unshaded basal portions was high, but the consumption of the basal portions decreased. Thus, although increased nutrient availability may reduce the cost of unshaded basal portions, may not promote *G. longituba* spread into shaded habitats through clonal integration, at least for the nutrient levels and shorter period set in our study. Clonal plants are the dominant species in natural habitats. Understanding the performance of clonal plants when they spread from unshaded to shaded habitats while maintaining ramets connections is helpful in predicting the distribution dynamics of plant populations and communities. However, the plant material used in this study originated from the same clone. Further experiments should be designed using clones of different genotypes or from various habitats to fully understand the effects of resource heterogeneity and clonal integration on the performance of *G. longituba*.

## Data availability statement

The original contributions presented in the study are included in the article/supplementary material. Further inquiries can be directed to the corresponding author.

## Author contributions

RZ: Formal analysis, writing–original draft, and writing—review and editing. Z-HC: Methodology, formal analysis, and writing—review and editing. Y-ML: Investigation and writing—review and editing. NW: Investigation and writing—review and editing. W-TC: Investigation and writing—review and editing. B-NZ: Formal analysis and writing—review and editing. CS: Conceptualization, methodology, formal analysis, writing—original draft, writing—review and editing, and supervision. All authors contributed to the article and approved the submitted version.
